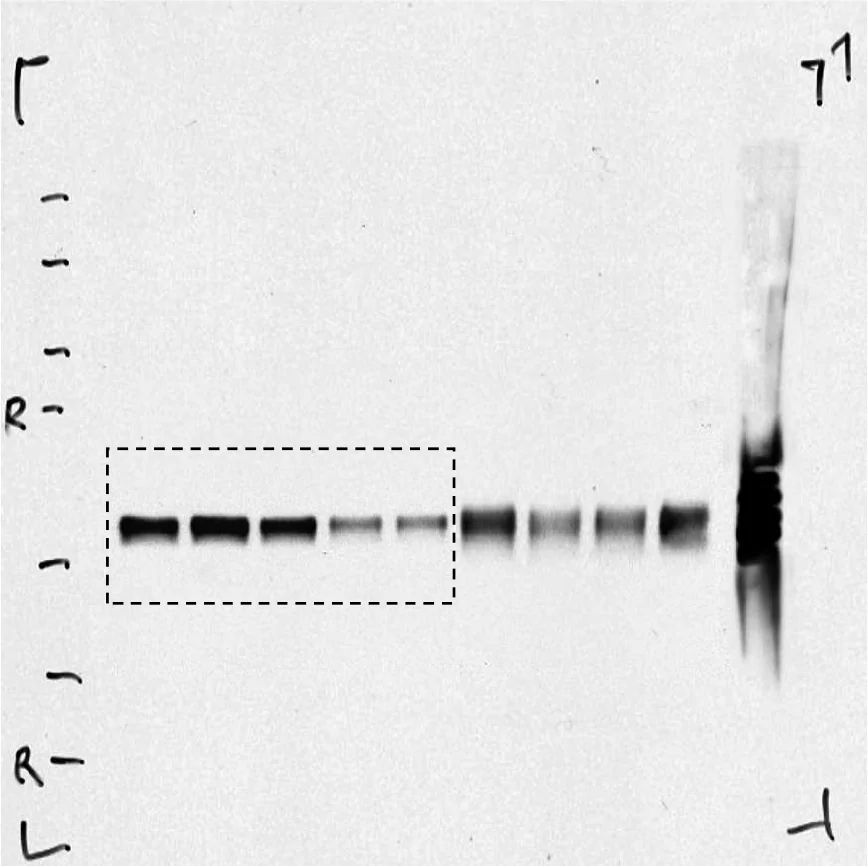# Correction: Targeted degradation of aberrant tau in frontotemporal dementia patient-derived neuronal cell models

**DOI:** 10.7554/eLife.113533

**Published:** 2026-10-23

**Authors:** M Catarina Silva, Fleur M Ferguson, Quan Cai, Katherine A Donovan, Ghata Nandi, Debasis Patnaik, Tinghu Zhang, Hai-Tsang Huang, Diane E Lucente, Bradford C Dickerson, Timothy J Mitchison, Eric S Fischer, Nathanael S Gray, Stephen J Haggarty

**Keywords:** Human

Silva MC, Ferguson FM, Cai Q, Donovan KA, Nandi G, Patnaik D, Zhang T, Huang H-T, Lucente DE, Dickerson BC, Mitchison TJ, Fischer ES, Gray NS, Haggarty SJ. 2019. Targeted degradation of aberrant tau in frontotemporal dementia patient-derived neuronal cell models. *eLife* **8**:e45457. doi: 10.7554/eLife.45457.

Published 25 March 2019

**Figure 1:**
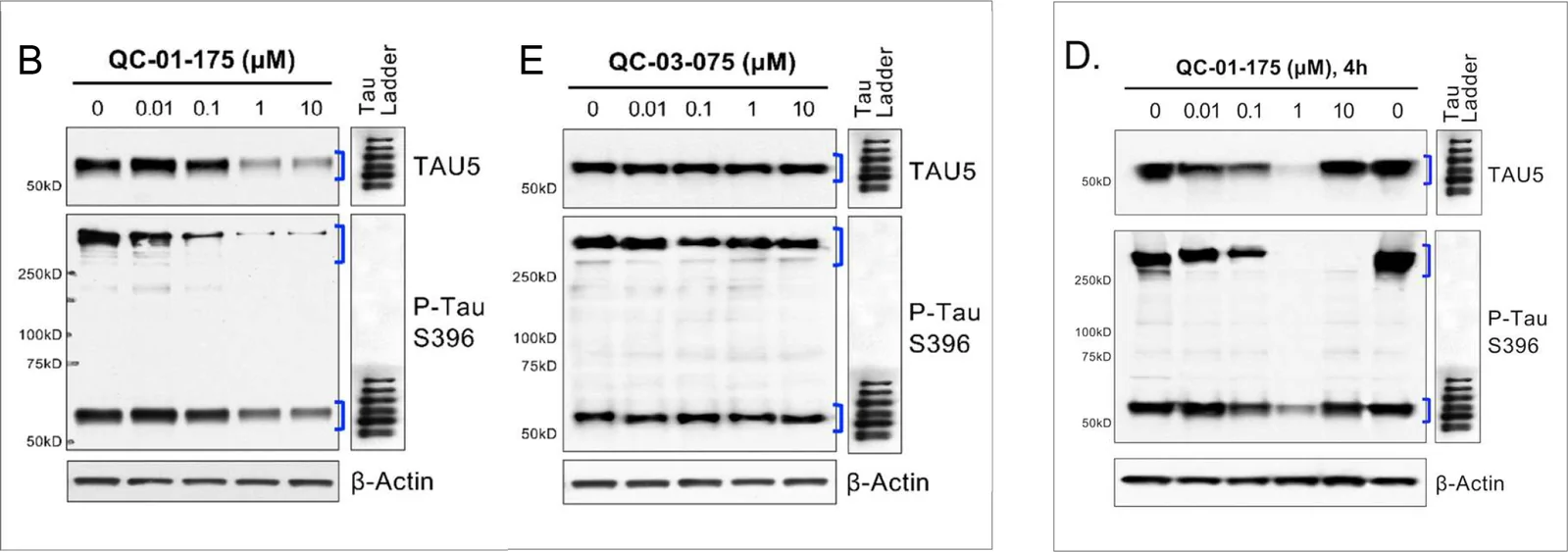


The authors were alerted to the issues addressed in this Correction via PubPeer and the eLife editorial office. The Tau ladder used throughout this study is an *E. coli*-derived recombinant total-tau protein standard representing six isoforms (0N3R, 0N4R, 1N3R, 1N4R, 2N3R, 2N4R), without post-translational modifications. It was included solely as a relative molecular-weight reference for the migration of monomeric tau, and not as quantified experimental data. Because the ladder is far more concentrated than the experimental samples, it requires a much shorter exposure and so is imaged separately from, but run alongside, the samples on the same gel.

Within Figure 2, the Tau ladder was run once, alongside the QC-01–175 samples (Figure 2B). The QC-03–075 samples (Figure 2E) were run on a separate gel in parallel, under identical conditions (same reagents, same run time), producing an equivalent migration pattern, so the same ladder image is shown next to both panels. The same ladder image is shown next to both the total Tau antibody (TAU5) and P-Tau S396 blots within each panel, although the phospho-tau antibody cannot detect the unmodified ladder – only a total-tau antibody can – so a P-Tau S396-detected version of the ladder image does not exist. The ladder was placed next to the blots solely as a relative reference for the migration of monomeric Tau (in this case also relative to higher MW oligomeric Tau). This same rationale applies to the panels in Figure 2–figure supplement 1 (A–I), Figure 3–figure supplement 1 (A–C), and Figure 5–figure supplement 1 (D). In each case, the ladder crop is positioned according to its migration relative to a co-run, commercially available dual-color pre-stained protein ladder (Bio-Rad Precision Plus Protein Dual Color Standards, 1610374). Because the gels were always run under identical conditions and reagents, the migration of the ladder is highly reproducible from gel to gel, so the same ladder crop could be positioned consistently across multiple panels without misrepresenting any individual blot’s migration pattern. The ladder is always framed and boxed separately from the sample lanes; it was not run individually for each experiment.

Manuscript Corrections

Corrections to the original manuscript include the added sentence to each relevant figure legend noting that the Tau ladder image is reused across the indicated panels. The original, unprocessed blot images corresponding to Figure 2 blots, where the Tau ladder was imaged, are now provided for comparison with the published, cropped panels.

Corrected Figure 2 legend text:

Representative western blots are shown (B, E) with mean densitometry quantification (bands corresponding to brackets) ± SEM (*n* = 3). The Tau ladder image shown in panels (B) and (E) is the same image, reused across both panels solely as a relative molecular-weight reference for monomeric tau migration; no data or conclusions are drawn from the ladder.

Originally published Figure 2 legend text:

Representative western blots are shown (B, E) with mean densitometry quantification (bands corresponding to brackets) ± SEM (*n* = 3).

Corrected Figure 2—figure supplement 1 legend text:

Figure 2—figure supplement 1. (..) Student T-test for each concentration/antibody relative to vehicle-treated, ^ns^p>0.05, *p≤0.01, **p≤0.001, ***p≤0.0001. Some blot images were cropped for the purpose of this figure only, to exclude samples not included in this manuscript (Silva and Ferguson, Manuscript in preparation, 2019), but all samples in each set were run in the same gel. The Tau ladder image shown in panels (A–I) is reused across these panels solely as a relative molecular-weight reference for monomeric tau migration; no data or conclusions are drawn from the ladder.

Originally published Figure 2—figure supplement 1 legend text:

Figure 2—figure supplement 1. (..) Student T-test for each concentration/antibody relative to vehicle-treated, ^ns^p>0.05, *p≤0.01, **p≤0.001, ***p≤0.0001. Some blot images were cropped for the purpose of this figure only, to exclude samples not included in this manuscript (Silva and Ferguson, Manuscript in preparation, 2019), but all samples in each set were run in the same gel.

Corrected Figure 3—figure supplement 1 legend text:

Figure 3—figure supplement 1. (...) Student T-test of compound-treated samples relative to vehicle-treated ***p<0.001, **p<0.01, *p<0.05, ^ns^p>0.05. The Tau ladder image shown in panels (A–C) is reused across these panels and the main figures as it is solely employed as a relative molecular-weight reference to identify monomeric tau migration; no data or conclusions are drawn from the ladder.

Originally published Figure 3—figure supplement 1 legend text:

Figure 3—figure supplement 1. (...) Student T-test of compound-treated samples relative to vehicle-treated ***p<0.001, **p<0.01, *p<0.05, ^ns^p>0.05.

Corrected Figure 5—figure supplement 1 legend text:

Figure 5—figure supplement 1. (...) Student T-test between each dose and vehicle-treated tau levels ^ns^p>0.05, *p<0.05, **p<0.01, ***p<0.001, ****p<0.0001. The Tau ladder image shown in panel (D) is reused from the same source image used elsewhere in this figure and in Figure 2, solely as a relative molecular-weight reference for monomeric tau migration; no data or conclusions are drawn from the ladder.

Originally published Figure 5—figure supplement 1 legend text:

Figure 5—figure supplement 1. (...) Student T-test between each dose and vehicle-treated tau levels ^ns^p>0.05, *p<0.05, **p<0.01, ***p<0.001, ****p<0.0001.

Additional Support Files/Data

Original, unprocessed, *low-exposure* blot used to capture the Tau ladder. This can be compared to the Tau ladder crops reused throughout the paper.

For completeness, the authors confirm that the Tau ladder image used in this article ***is distinct from the Tau ladder image presented in any other previously or subsequently published figure***, which was verified through direct review of the original, unprocessed blot images provided to the eLIFE editorial office.

**Figure 2:**
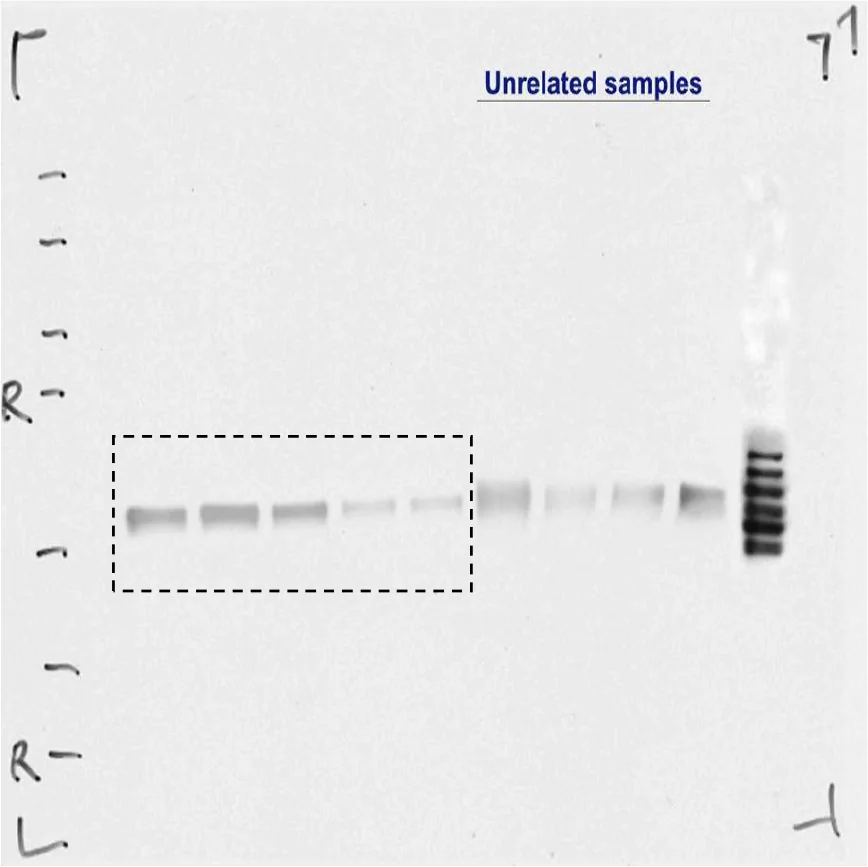


Original, unprocessed, *high-exposure* blot showing the monomeric tau bands, to be compared with the published, cropped panel in Figure 2B.

**Figure 3:**