# Supplementary figures and images for: Targeted degradation of aberrant tau in frontotemporal dementia patient-derived neuronal cell models
Source: eLife. 2019 Mar 25;8:e45457. doi: 10.7554/eLife.45457 (PMC6450673; doi:10.7554/eLife.45457)

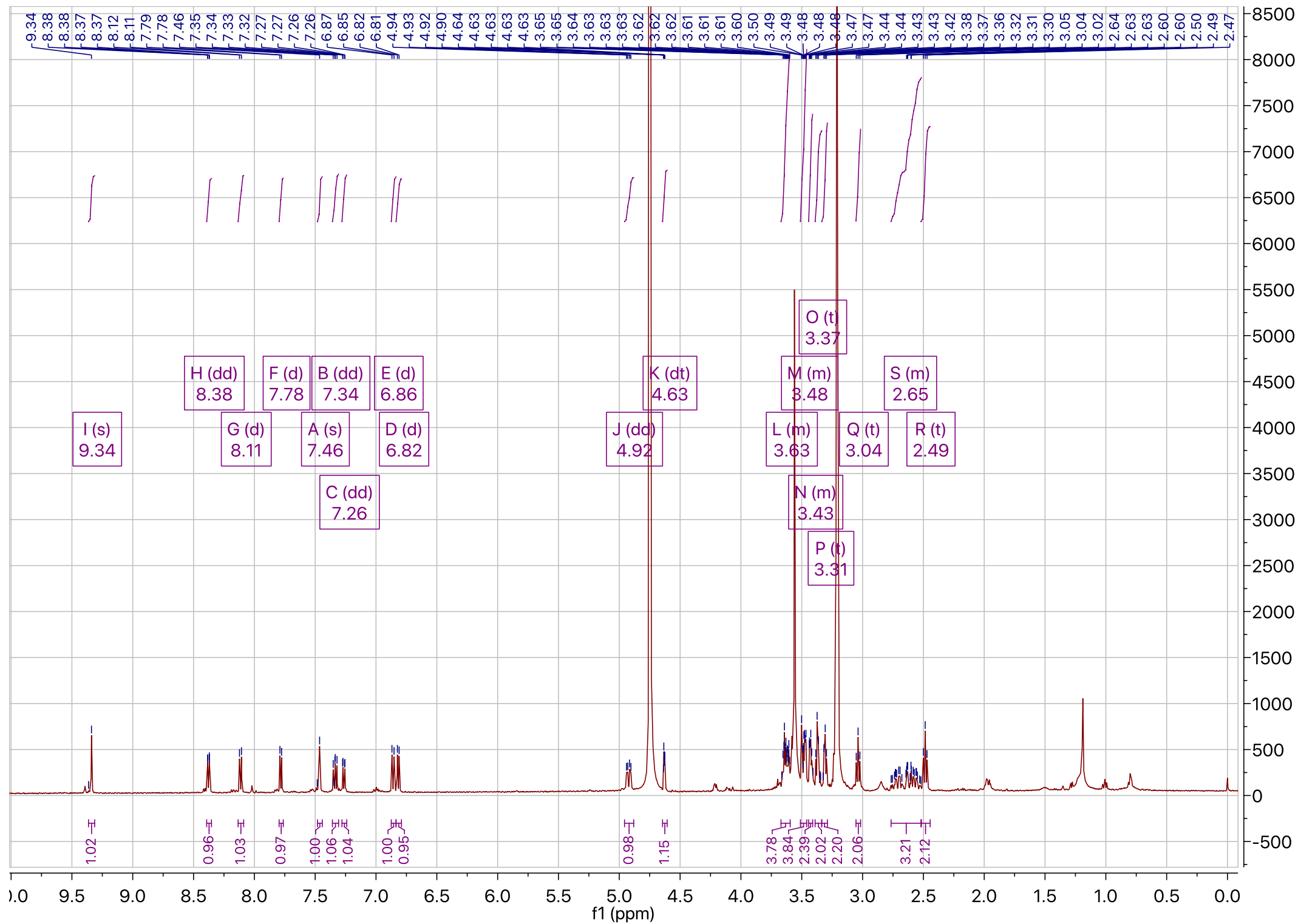

Supplement: Supplementary file 1. [file elife-45457-supp1.pdf]

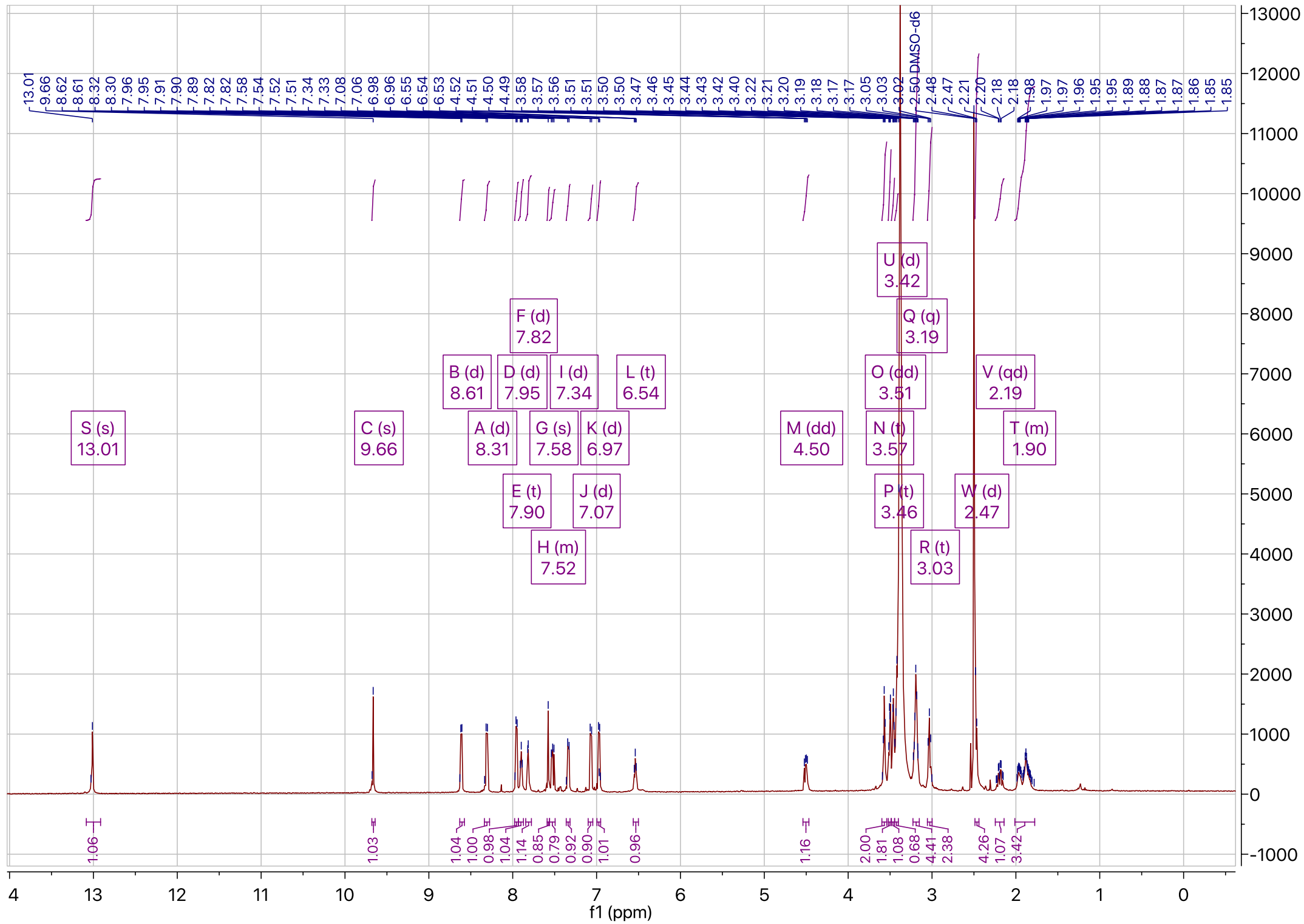

Supplement: Supplementary file 2. [file elife-45457-supp2.pdf]

05-Nov-2018 14:37:18

QC-01-175-1

3: Diode Array  
Range: 1.403e+2

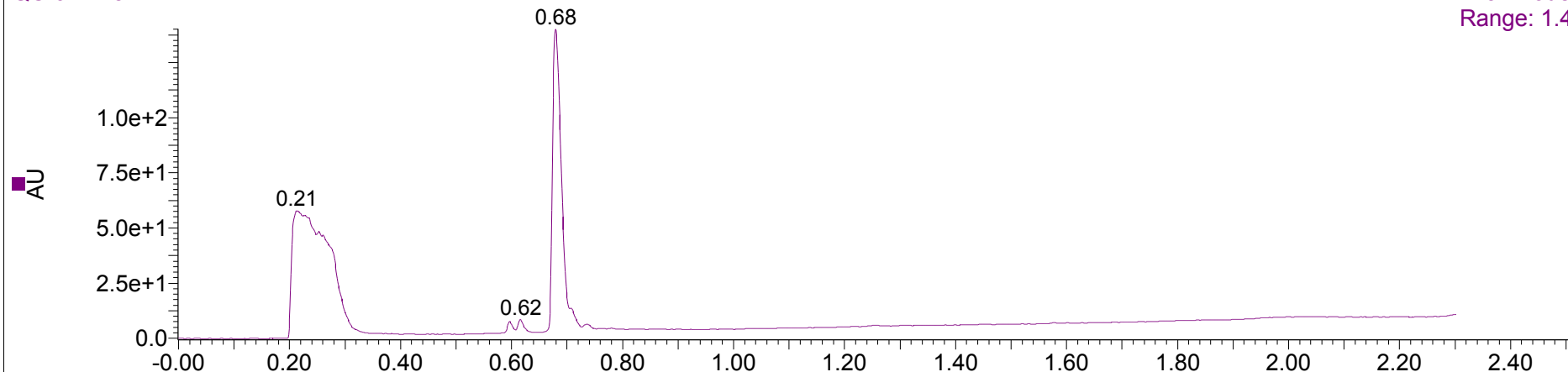

QC-01-175-1

2: Scan ES-  
TIC  
4.71e6

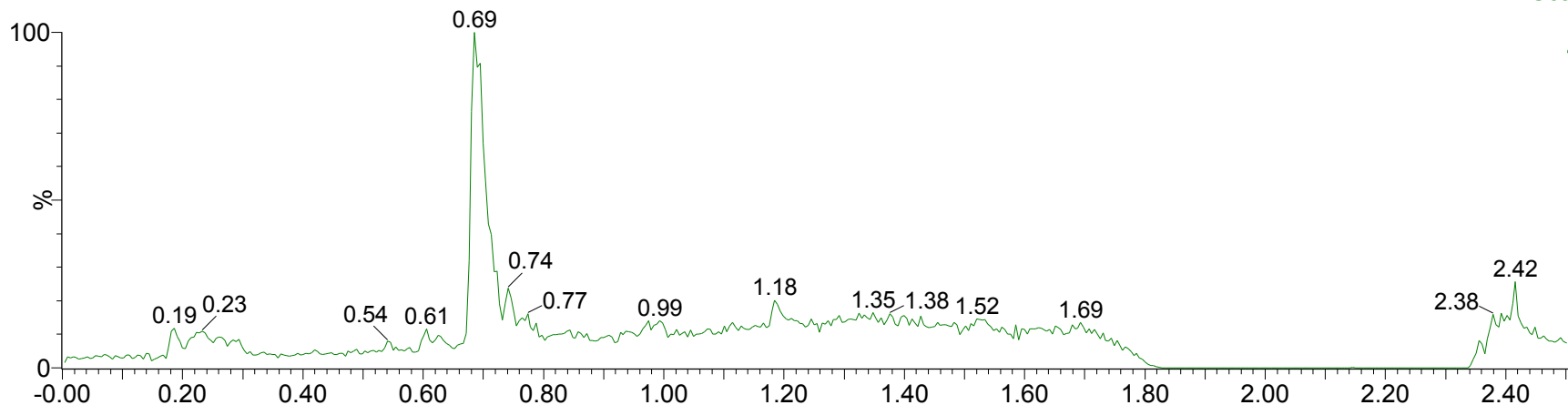

QC-01-175-1

1: Scan ES+  
TIC  
9.93e7

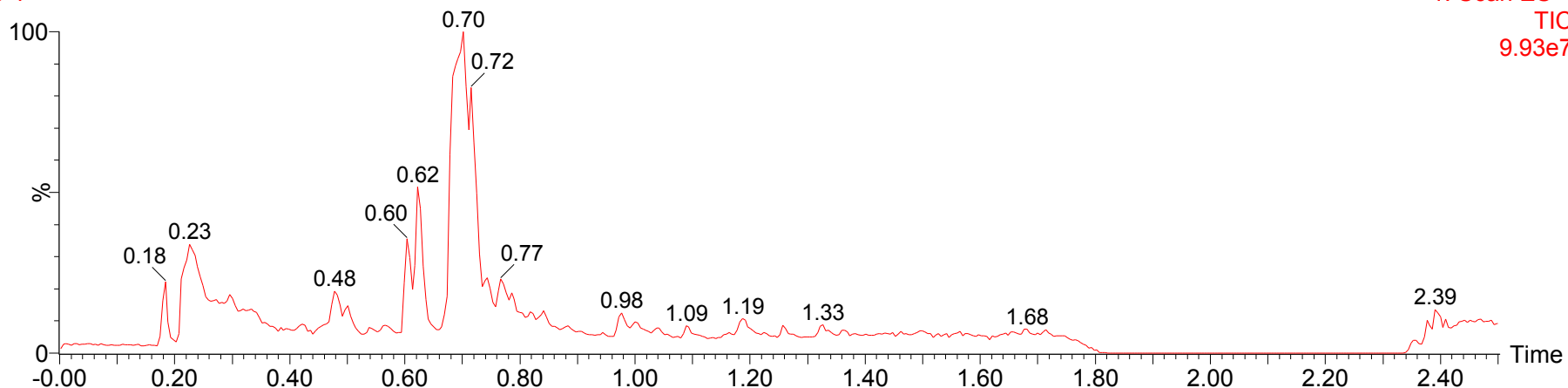

QC-01-175-1 149 (0.692)

1: Scan ES+  
5.14e7

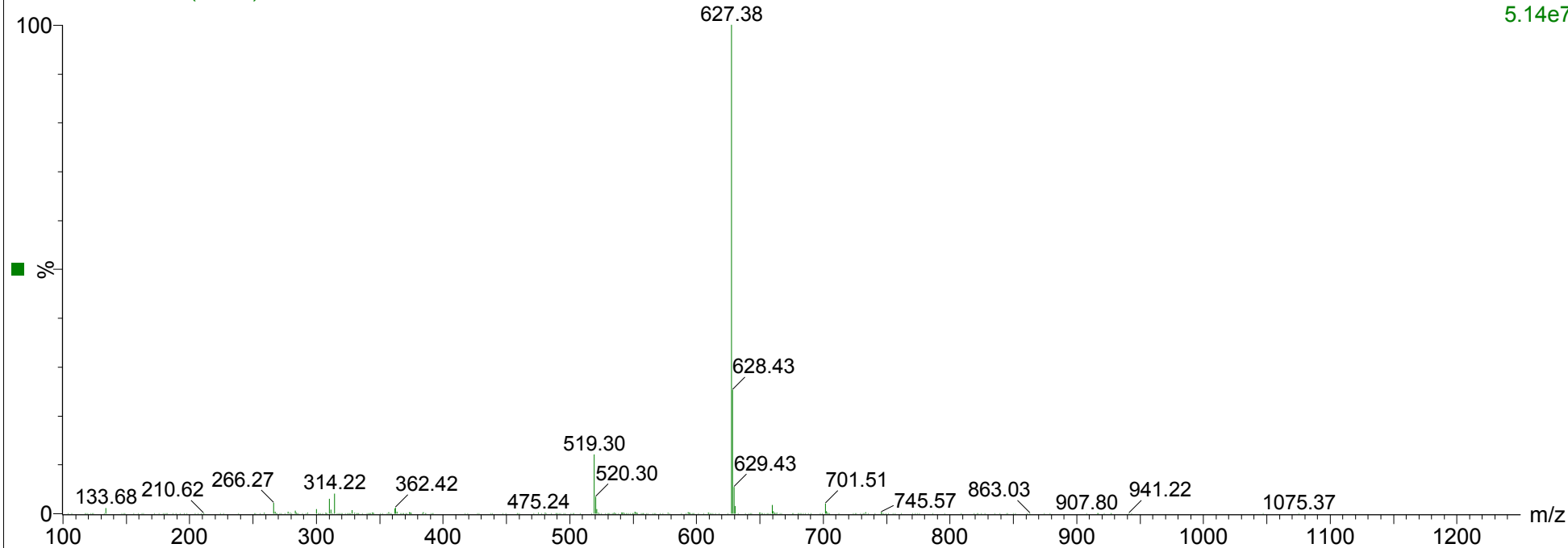

QC-01-175-1 151 (0.704)

2: Scan ES-  
1.16e6

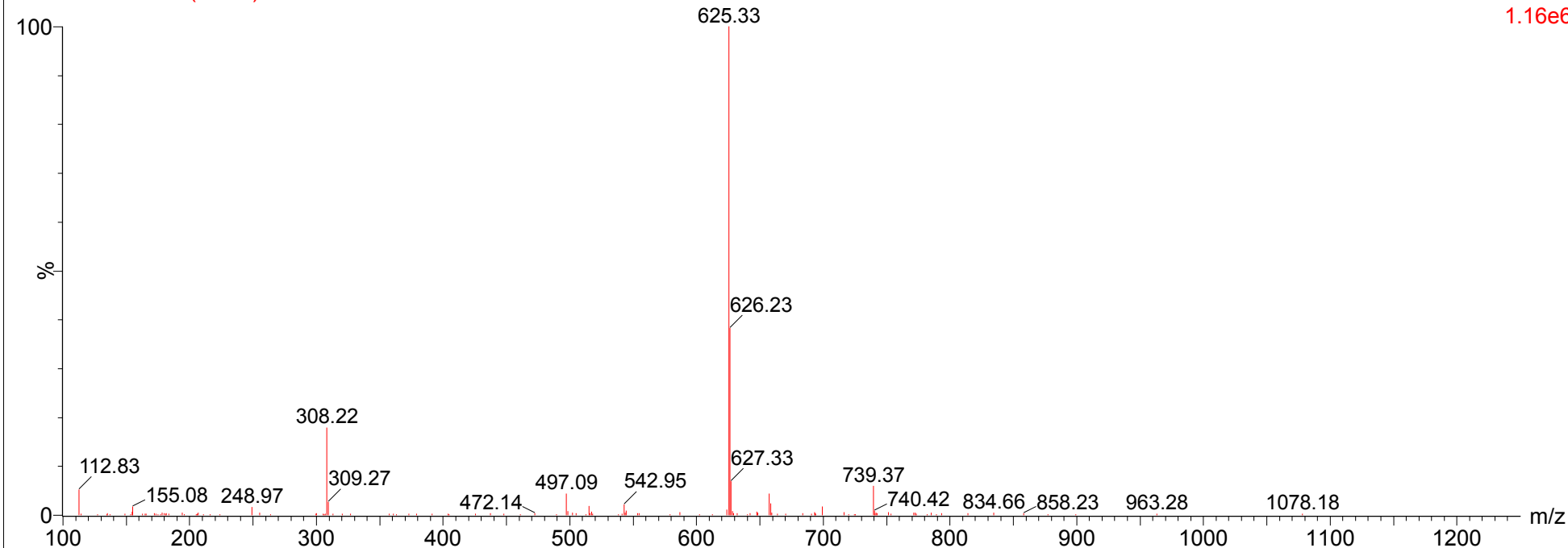

Supplement: Supplementary file 3. [file elife-45457-supp3.pdf]

05-Mar-2019 14:14:27

QC-03-075-1

3: Diode Array  
Range: 2.939e+2

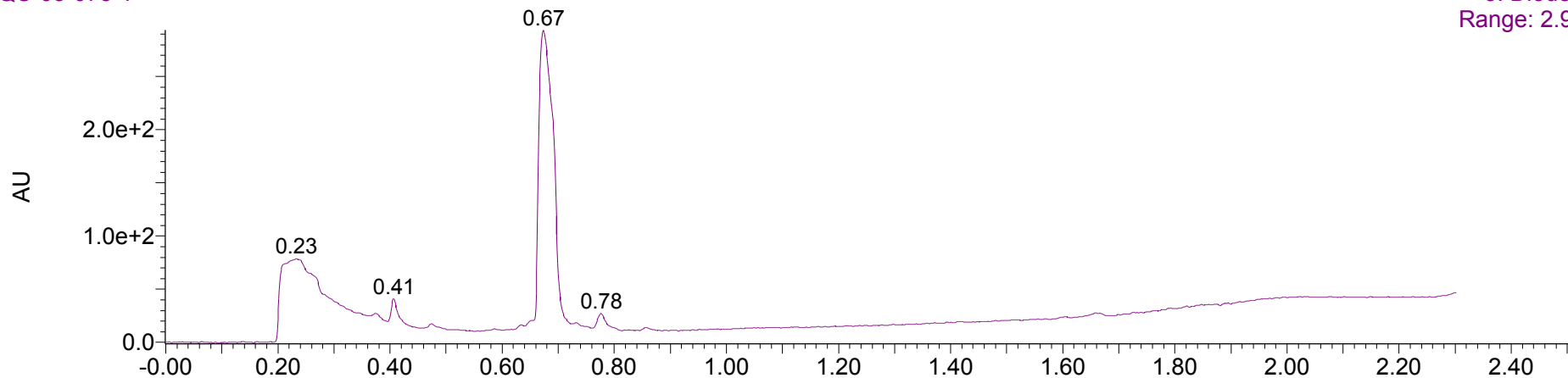

QC-03-075-1

2: Scan ES-  
TIC  
5.94e5

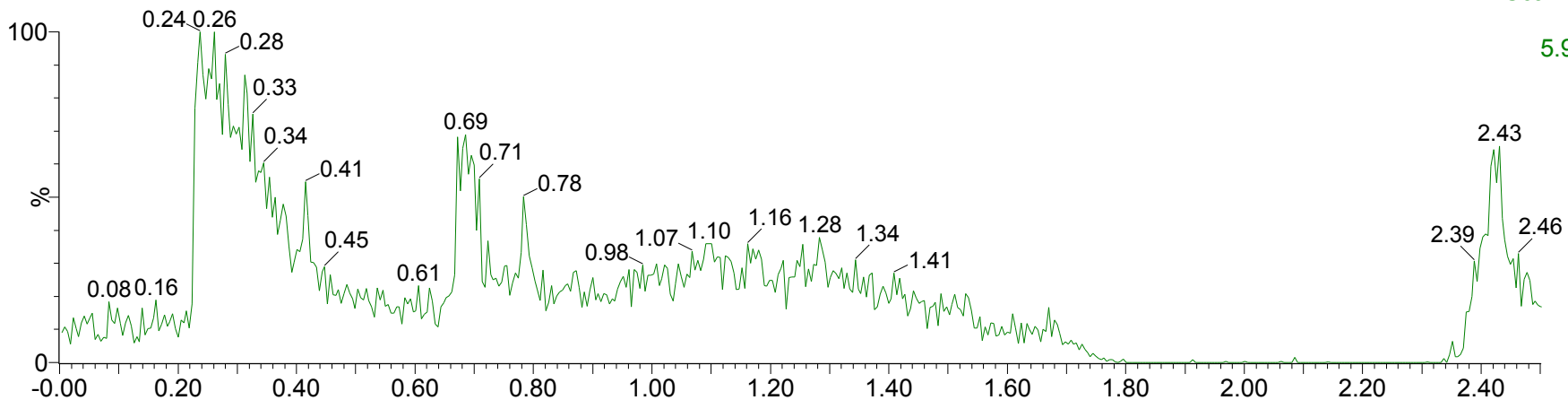

QC-03-075-1

1: Scan ES+  
TIC  
1.44e7

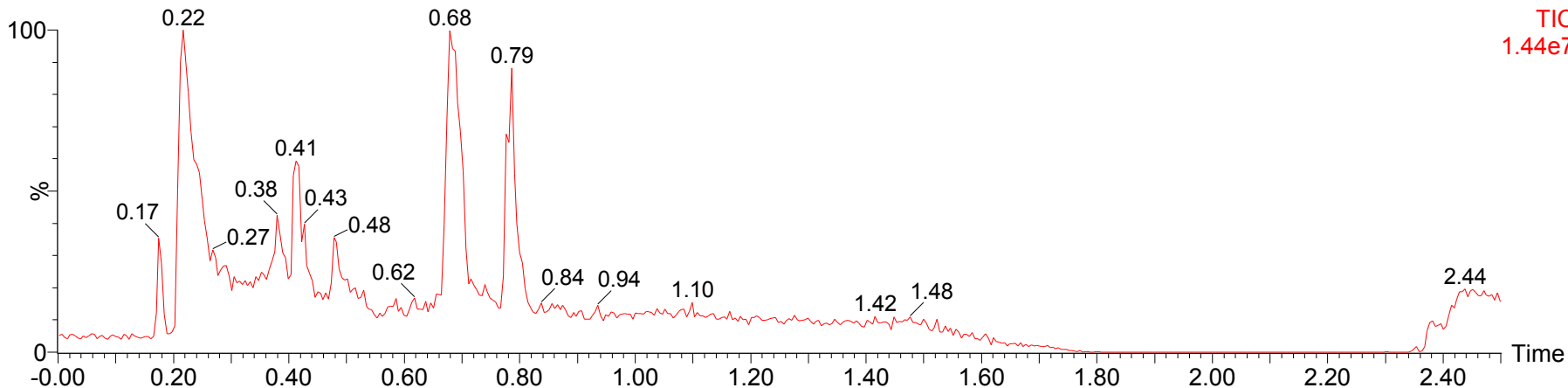

QC-03-075-1 147 (0.683)

1: Scan ES+  
1.03e7

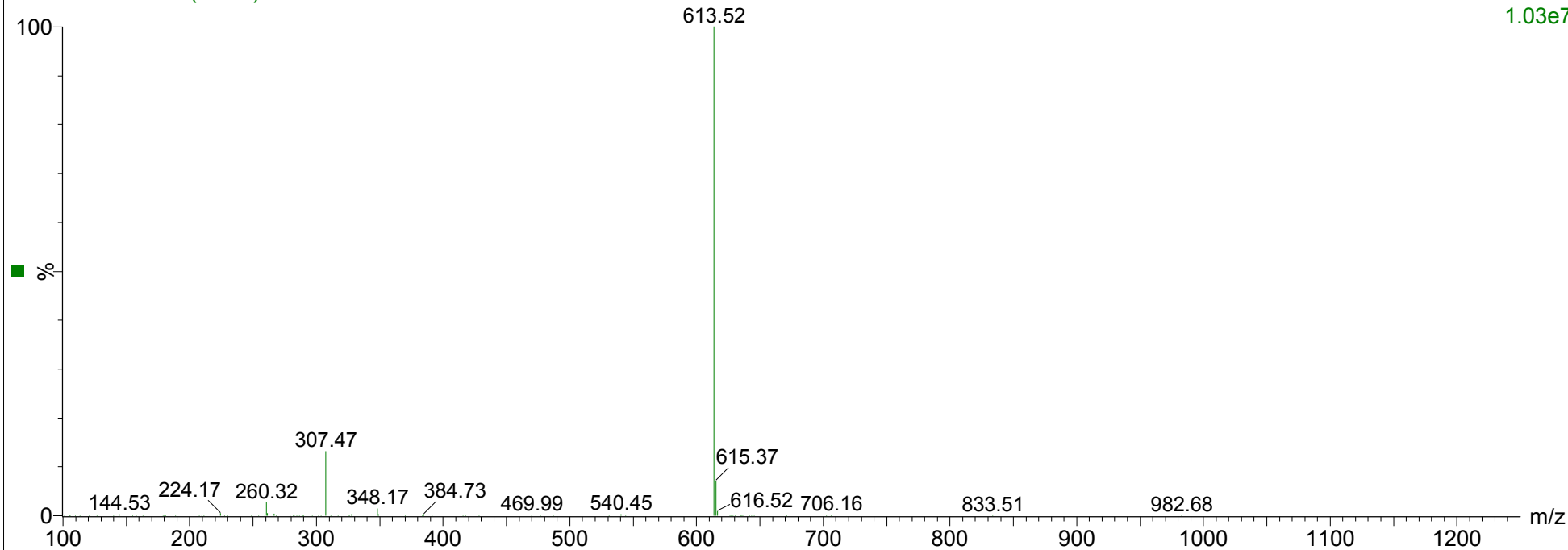

QC-03-075-1 146 (0.681)

2: Scan ES-  
2.58e5

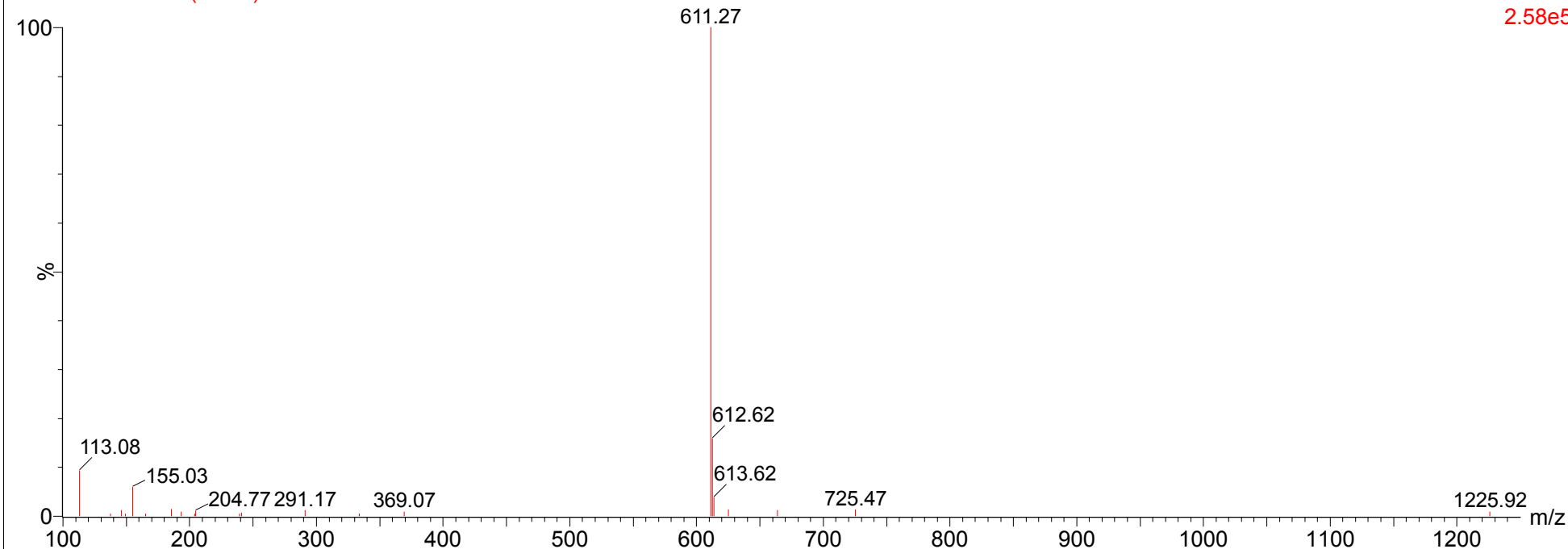

Supplement: Supplementary file 4. [file elife-45457-supp4.pdf]
